# Assessment of the Anti-Biofilm Effect of Cefiderocol Against 28 Clinical Strains of Multidrug-Resistant Gram-Negative Bacilli

**DOI:** 10.3390/antibiotics14080738

**Published:** 2025-07-23

**Authors:** Marta Díaz-Navarro, Emilia Cercenado, Andrés Visedo, Mercedes Marín, Marina Machado, Álvaro Irigoyen-von-Sierakowski, Belén Loeches, Juana Cacho-Calvo, Julio García-Rodríguez, Enea G. Di Domenico, Patricia Muñoz, María Guembe

**Affiliations:** 1 Department of Clinical Microbiology and Infectious Diseases, Hospital General Universitario Gregorio Marañón, 28007 Madrid, Spain; maartadn@gmail.com (M.D.-N.); ecercenado@gmail.com (E.C.); andresvsg777@gmail.com (A.V.); mercedes.marinar@salud.madrid.org (M.M.); marinamachadov@gmail.com (M.M.); airigoyenvs@gmail.com (Á.I.-v.-S.); pmunoz@hggm.es (P.M.); 2 Instituto de Investigación Sanitaria Gregorio Marañón, 28009 Madrid, Spain; 3 School of Medicine, Universidad Complutense de Madrid, 28040 Madrid, Spain; 4 CIBER Enfermedades Respiratorias—CIBERES (CB06/06/0058), 28029 Madrid, Spain; 5 Department of Clinical Microbiology, Hospital Universitario La Paz, 28046 Madrid, Spain; bloeches@yahoo.es (B.L.); juanabegona.cacho@salud.madrid.org (J.C.-C.); juliogarciarodriguez@gmail.com (J.G.-R.); 6 Microbiology and Virology, San Gallicano Dermatological Institute, IRCCS, 00144 Rome, Italy; enea.didomenico@ifo.gov.it

**Keywords:** cefiderocol, biofilm, MIC, MBIC, multidrug-resistant Gram-negative bacilli, extracellular matrix

## Abstract

**Objectives**: Cefideroccol (FDC) is a siderophore cephalosporin with potent antibacterial activity against a wide range of Gram-negative multidrug-resistant (MDR) microorganisms. We investigated the anti-biofilm capacity of FDC against clinical strains. **Methods**: This multicenter study was conducted on 28 selected strains of MDR Gram-negative bacilli isolated from clinical samples of *Pseudomonas aeruginosa* (n = 5), *Acinetobacter baumannii* (n = 11), and *Klebsiella pneumoniae* (n = 12). We first determined the minimum inhibitory concentration (MIC) of each strain using the microdilution method. We also defined the minimum biofilm inhibitory concentration (MBIC) as a ≥50% reduction in tetrazolium salt (XTT) (as recommended in the 2017 Spanish Microbiology Protocols [SEIMC] for the microbiological diagnosis of infections related to the formation of biofilms). We also analyzed the reduction in the following biofilm variables after an 8 mg/mL FDC treatment: the CFU count, the cell viability, the biomass, the metabolic activity, and extracellular α or β polysaccharides. **Results**: The MIC_50_ and MBIC_50_ of FDC were 0.5 mg/L and 64 mg/L, respectively. We observed a mean (SD) fold increase in the susceptibility to FDC between planktonic and sessile cells for *P. aeruginosa*, *A. baumannii*, and *K. pneumoniae* of 9.60 (0.55), 6.27 (2.28), and 6.25 (2.80), respectively. When 8 mg/mL of FDC was tested, we observed that the best median (IQR) percentage reductions were obtained for cell viability and the extracellular matrix (73.1 [12.4–86.5] and 79.5 [37.3–95.5], respectively), particularly for *P. aeruginosa*. The lowest percentage reduction rates were those obtained for biomass. **Conclusions**: We demonstrated that the susceptibility to FDC was significantly reduced when strains were in a biofilm state. The best percentage reduction rates for all biofilm-defining variables were observed for *P. aeruginosa*. Our results need to be validated using a larger collection of clinical samples.

## 1. Introduction

Biofilms play an important role in the pathogenesis of most healthcare-related infections occurring on tissues and on medical devices. Such infections induce chronic inflammation, and therefore, they hamper the response to antimicrobial agents [1,2]. The microbiome is also a major component in the pathogenesis and progression of infectious diseases [3,4,5]. Specific multidrug-resistant (MDR) microorganisms, such as Gram-negative bacilli, are becoming increasingly frequent and exert a major impact on the clinical outcome and patient management, especially for critical care patients [1,6,7,8]. Moreover, MDR microorganisms behave differently according to the species studied. For example, two distinct biofilm morphotypes of *Achromobacter* spp. have been identified in patients with cystic fibrosis. These morphotypes were associated with varying levels of antibiotic tolerance. Interestingly, *P. aeruginosa* strains isolated from cystic fibrosis patients exhibited a lower in vivo virulence compared to strains from non-cystic fibrosis sources [9,10].

Cefiderocol (FDC) is a new broad-spectrum siderophore cephalosporin with marked activity against a broad range of Gram-negative pathogens, including MDR strains [11,12,13,14]. As siderophore antibiotics bind ferric iron and use iron transporters to cross the cell membrane, FDC has a huge advantage against biofilms, where antibiotic resistance is high, but iron scavenging is important [15,16]. FDC has proven to be a promising agent, alone or in combination with other antibiotics, for treating healthcare-related infections caused by MDR Gram-negative bacteria, such as pneumonia and even infective endocarditis [17,18,19]. However, data regarding its efficacy against bacterial biofilms are somewhat scarce [16,20].

On this basis, we aimed to assess the anti-biofilm activity of FDC against a wide range of Gram-negative pathogens by studying different biofilm-defining variables in an in vitro model.

## 2. Results

### 2.1. MIC and MBIC of FDC

**Figure 1:**
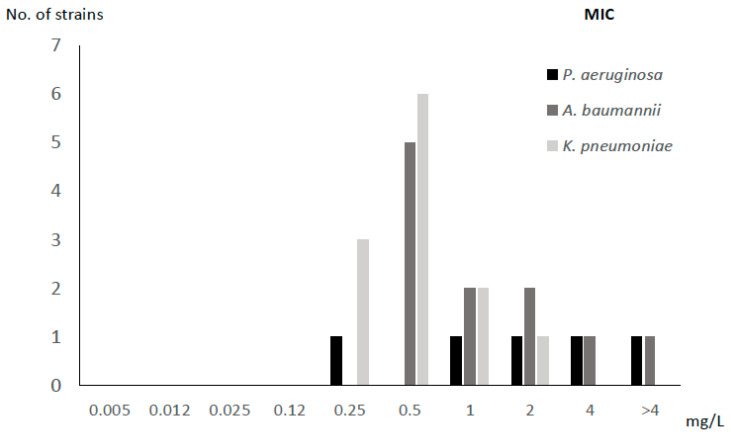
MIC for cefiderocol according to bacterial species.

**Figure 2:**
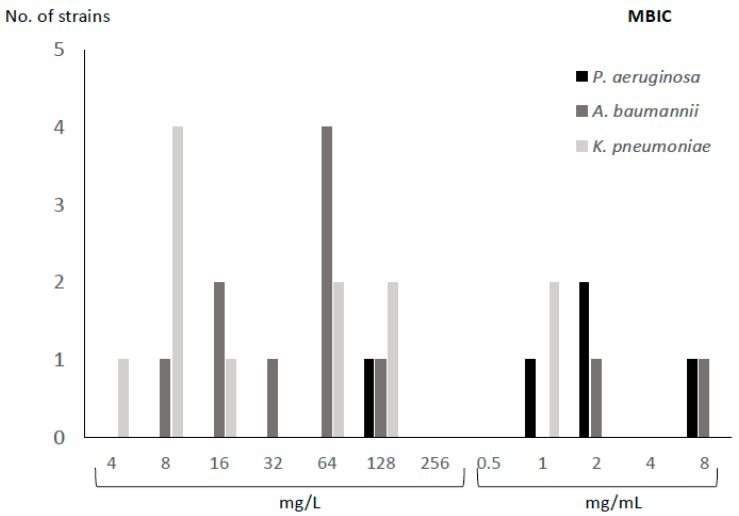
MBIC for cefiderocol according to bacterial species.

The MIC_50_ and MBIC_50_ of FDC were 0.5 mg/L and 64 mg/L, respectively. According to species, the mean (SD) fold increase in the susceptibility to FDC between planktonic and sessile cells for *P. aeruginosa*, *A. baumannii*, and *K. pneumoniae* was 9.60 (0.55), 6.27 (2.28), and 6.25 (2.80), respectively (Table 1, Figure 1 and Figure 2).

With respect to the MBIC, all strains ranged between 4 and 8000 mg/L. The highest MBIC values were observed for *P. aeruginosa*, as the lowest concentration at which biofilm was inhibited by >50% was 128 mg/L, compared to *A. baumannii* and *K. pneumoniae*, for which the values were 8 and 4 mg/L, respectively. Only two strains (one *P. aeruginosa* and one *A. baumannii*) had an MBIC of 8000 mg/L (Table 1).

### 2.2. Anti-Biofilm Testing of an 8 mg/mL FDC Concentration

**Figure 3:**
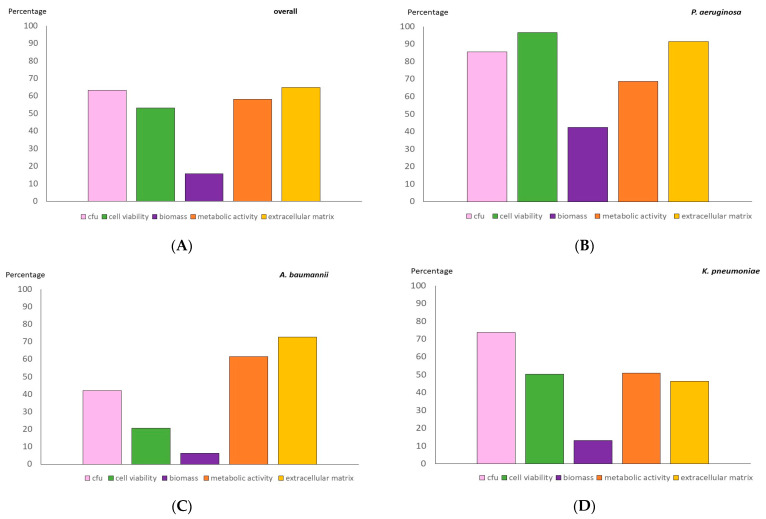
Mean percentage reduction in log CFU counts, cell viability, biomass, metabolic activity, and extracellular matrix after 48 h treatment of 24 h biofilms with 8 mg/mL of cefiderocol. (**A**) overall, (**B**) *Pseudomonas aeruginosa*, (**C**) *Acinetobacter baumannii*, and (**D**) *Klebsiella pneumoniae*.

The median (IQR) percentage reduction in the log CFU/mL, the cell viability, the biomass, the metabolic activity, and the extracellular matrix was as follows: 67.8 (47.7–80.2), 73.1 (12.4–86.5), 0.0 (0.0–28.9), 59.3 (28.1–97.4), and 79.5 (37.3–95.5), with the best reductions observed for *P. aeruginosa*. The poorest results were recorded for *A. baumannii* in almost all the variables tested (Table 2 and Figure 3). The biomass reduction was the only variable whose results were not consistent with those of the others (*p* < 0.001).

For *P. aeruginosa* in particular, the results were significantly better for the median (IQR) reduction in the log CFU/mL and cell viability than for *A. baumannii* (86.7 [73.8–96.7] and 93.2 [86.5–98.0] vs. 43.0 [20.7–53.2], respectively; *p* = 0.009 and *p* = 0.029). The same was true for the median (IQR) reduction in the cell viability rate compared to *K. pneumoniae* (93.2 [86.5–98.0] vs. 6.3 [14.3–81.5], *p* = 0.008). In addition, the results were significantly better for *K. pneumoniae* in terms of the median (IQR) reduction in the log CFU/mL than for *A. baumannii* (75.6 [63.1–80.2] vs. 43.0 [20.7–53.2], *p* = 0.002) (Table 3).

**Figure 4:**
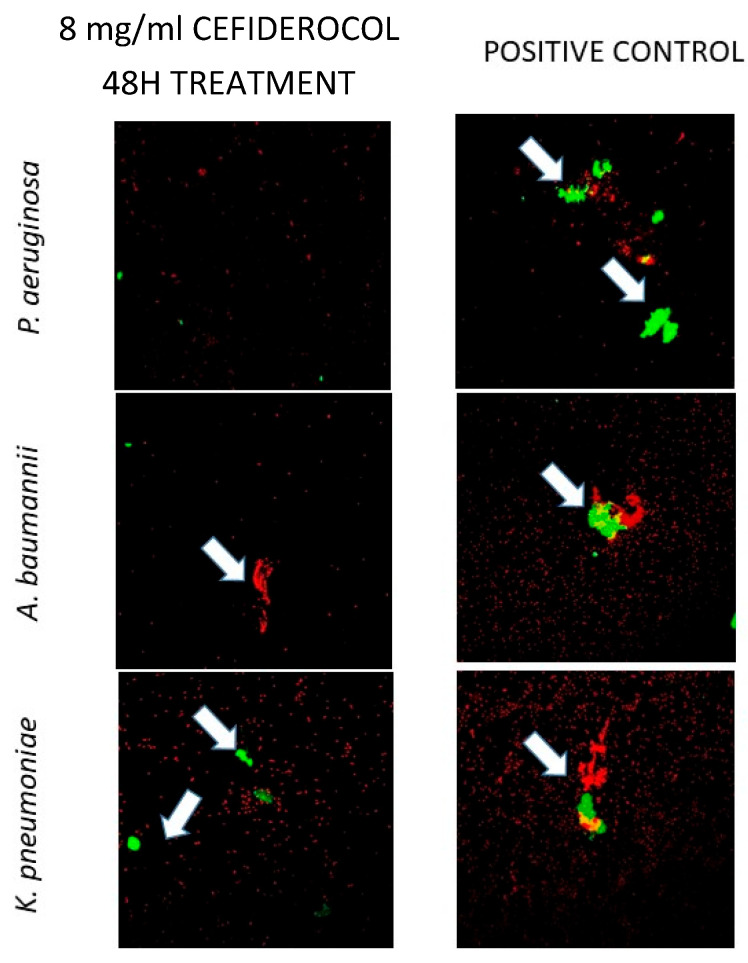
Representative images obtained by confocal laser scanning microscopy of 24 h biofilms stained with GS-II by species in cefiderocol-treated and untreated groups.

Figure 4 shows the difference not only in the area occupied by polysaccharides from the extracellular matrix (irregular areas occupying more space stained with red–green), but also in the number of bacteria (small bacilli stained in red) from the 24 h biofilms treated and not treated with 8000 mg/L of CFD for each species (one strain per species).

The analysis was performed using *Griffonia simplicifolia* Lectin II (GS-II) conjugated with Alexa fluor 488 dye after a 1 h application to the biofilms formed in the glass slides using 0.5% Triton-X 100 and 4% formaldehyde. The stained samples were examined using a Leica TCS SPE confocal fluorescence microscope (Leica Geosystems AG, Heerbrugg, Switzerland). Images of a representative strain for each species are shown. The percentage occupation area of GS-II was calculated. White arrows indicate the extracellular matrix.

## 3. Discussion

Healthcare-associated infections caused by Gram-negative bacteria characterized by a difficult-to-treat resistance phenotype are associated with worse clinical outcomes and pose daily therapeutic challenges, especially in critical care patients [18,21,22]. Moreover, biofilm reduction is clinically significant in managing MDR infections because biofilms act as a protective barrier, shielding bacteria from antibiotics and the immune system. So, it is important to enhance the disruption of biofilms for better antimicrobial penetration and bacterial susceptibility, and to reduce the risk of chronic or recurrent infections. Effective biofilm control also enhances wound healing, reduces complications in medical implants, and improves patient outcomes, making it a critical strategy in combating MDR pathogens.

Therefore, new antibiotics to treat MDR bacterial biofilm-mediated infections must be developed. FDC is an iron-carrying cephalosporin that enters the bacterial periplasm as a “Trojan horse”. Since it is highly active against MDR Enterobacteriaceae and non-fermentative bacteria, it seems to be a promising alternative for the treatment of adults and children with treatment-refractory infections [18], as it retains activity by achieving higher intracellular concentrations and maintaining stability against a broad range of β-lactamases. This makes it a valuable therapeutic option in clinical settings for managing persistent MDR infections where conventional antibiotics often fail.

However, the emergence of resistance should not be overlooked, as reported for specific carbapenem-resistant Gram-negative bacterial phenotypes [23]. In the specific case of *K. pneumoniae*, a potential negative feedback effect was recently reported between the reduction in the biofilm formation ability and siderophore transporter proteins [15]. This may be a crucial factor when *K. pneumoniae* simultaneously reduces biofilm formation and acquires resistance to FDC.

We demonstrated that, as expected, the susceptibility to FDC was significantly reduced when the strains were in a biofilm state, with a concentration of at least 8000 mg/L being required to ensure adequate eradication. We observed a wide range of MBIC values across different strains, which may be explained by several potential factors contributing to that, such as phenotypic or genetic factors related to biofilm and extracellular matrix production. Our study is the first to assess the efficacy of FDC against bacterial biofilms based on five biofilm-defining variables, and, as the results obtained for the various techniques do not necessarily have to coincide, it is desirable to interpret them together.

Only two previous studies have addressed the MBIC of FDC against clinical isolates by assessing only the biomass or cell viability by CFU counts. The study by Pybus et al. [16] focused mainly on the possible differences in the MIC and MBICs obtained using either iron-depleted cation-adjusted Mueller–Hinton broth or Mueller–Hinton II broth in four to five strains of six MDR Gram-negative genera. Unlike the impact of media on the MIC, where the FDC MIC_90_ values in Mueller–Hinton II broth were generally 2-fold higher in most cases, no differences in the average biofilm reduction rates were reported for *P. aeruginosa* or *K. pneumoniae* strains when one or the other medium was used. Although the authors only tested up to a concentration of 32 mg/L, they also observed discrepancies between the MIC and MBIC values, as did we. The other study is that of Ferretti et al. [20], who analyzed 24 h biofilms of 10 *P. aeruginosa* clinical isolates from infected implants and blood that were treated with FDC, alone or in combination with imipenem, testing up to a concentration of 512 mg/L. The authors assessed the MBIC, defined as the lowest concentration for a reduction of at least 3 log 10 CFU/mL, and used scanning electron microscopy, thus demonstrating a remarkable reduction in the CFU count and the inhibition of bacterial division, especially when the two drugs were combined. One of the main limitations of both studies is that they were based on the methods used. Biomass is not the most reliable biofilm quality by which to quantify reduction, because it does not differentiate between viable and nonviable cells [24], as demonstrated in our study, where the overall median percentage reduction in biomass was 0.0 (0.0–28.9). In addition, while the CFU count does not necessarily represent viability, it does indicate that viable and nonculturable cells are present [25], with the result that methods other than conventional culture need to be applied. We solved these problems by assessing the FDC activity using five different methods. The concordance was very good for all of them except biomass, and, in the case of *A. baumannii*, biomass and cell viability. When we analyzed each species individually, we observed that the best reductions were for *P. aeruginosa* in all the variables tested compared to *K. pneumoniae* and *A. baumannii*, with this difference being statistically significant for both species in cell viability and for *A. baumannii* in cell viability and CFU counts.

One of the main limitations of the study is that, although our pilot experiment with *P. aeruginosa* ATCC27853 and previous studies demonstrated that no significant differences in the MBIC were observed depending on the media used [16], we only tested FDC prepared in Mueller–Hinton broth without an iron addition. However, as it was applied to both the MIC and the MBIC, the results are reliable. The other main limitation of the study is its relatively low sample size, so studies including a large sample size and evaluating the mechanism from which biofilms affect antimicrobial susceptibility are further needed.

## 4. Materials and Methods

This multicenter study (2 centers in Spain and 1 center in Italy) was conducted on 28 selected MDR Gram-negative bacillus strains of the following species: *Pseudomonas aeruginosa* (n = 5), *Acinetobacter baumannii* (n = 11), and *Klebsiella pneumoniae* (n = 12) isolated from clinical samples (blood, n = 16; rectal exudate, n = 4; abscess, n = 3; bronchial aspirate, n = 3; bronchoalveolar lavage, n = 1; pharyngeal exudate, n = 1).

We first determined the minimum inhibitory concentration (MIC) of FDC for each strain using the microdilution method (serial dilutions from 0.005 mg/L to 4 mg/L) [26]. We also defined the minimum biofilm inhibitory concentration (MBIC) using 5 × 10^5^ colony-forming units (CFU)/mL bacterial suspensions in Mueller–Hinton broth (Sigma-Aldrich^®^, Darmstadt, Germany) that were inoculated into 96-well plates, which were incubated for 24 h at 37 °C. Next, the wells were washed with phosphate-buffered saline (PBS) (Sigma-Aldrich^®^, Germany) and treated with either FDC solution (serial dilutions from 0.004 mg/L to 8000 mg/L) or sterile saline (positive controls). The plates were incubated for 24 h at 37 °C, washed with PBS, and allowed to dry. We then added 100 µL of tetrazolium salt (XTT) (menadione, 1:1000) (Sigma-Aldrich^®^, Germany) to each well and incubated the plates for 2 h at 37 °C in darkness. The absorbance of each well was measured at 492 nm using a spectrophotometer, and the percentage reduction in the metabolic activity of the treated wells with respect to the control wells was calculated. We defined the MBIC as the lowest concentration that enabled a ≥50% reduction in the absorbance of XTT [27].

We selected a final FDC concentration of 8000 mg/L, which was the concentration at which all strains showed a ≥50% reduction in biofilm, and tested it against 24 h biofilms to assess the percentage reduction in the following biofilm-defining variables: the log CFU, the cell viability, the biomass, the metabolic activity, and the extracellular matrix.

The percentage reduction was calculated as (1 − [value_CFD_/value_positive control_]) × 100.

A pilot study using a *P. aeruginosa* ATCC27853 strain was performed to confirm that the MIC value obtained by our laboratory method (0.5 mg/L) was similar to that reported by the Clinical & Laboratory Standards Institute (0.06–0.5 mg/L).

### 4.1. Biofilm Procedure

Biofilms were formed by inoculating a fresh 24 h culture of each strain into 20 mL of a broth heart infusion and incubating at 37 °C in an orbital shaker for 24 h. The inocula were then washed in 3 centrifuge–resuspension cycles with PBS, and the pellets were resuspended in 10 mL of a brain heart infusion broth. These suspensions were adjusted to 0.5 McFarland turbidity using a turbidimeter, and 100 μL was inoculated in a non-tissue-prepped 96-well plate (for the GS-II-Alexa fluor 488, we used coverslips on a 24-well plate). After 24 h of biofilm formation at 37 °C, the plates were washed 3 times with PBS followed by a 48 h treatment with 100 μL of an 8000 mg/L FDC solution (or saline for the positive controls). The wells were then washed 3 times with PBS to remove unattached cells.

### 4.2. CFU Counts and Cell Viability

An analysis was performed by scraping the biofilm at the bottom of the well and resuspending it in PBS, which was used to calculate the log CFU/mL counts by a conventional culture of serial dilutions on blood agar plates and the cell viability rate by flow cytometry (Gallios, Beckman Coulter, BioRad, Madrid, Spain). Flow cytometry images were acquired by adjusting the settings to include only singlets in the analysis. The resulting data were analyzed using the Kaluza Analysis 2.1. software application.

### 4.3. Biomass and Metabolic Activity

The analysis was performed using the crystal violet stain and XTT to assess the median (IQR) absorbance values in the spectrophotometer for, respectively, treated biofilms and positive controls.

In the CV assay, bacteria were cultured in microtiter plates under biofilm-promoting conditions. After incubation, non-adherent cells were washed away, and the remaining biofilm was stained with CV dye, which binds to biomass (cells and extracellular matrix). Excess dye was rinsed off, and the retained stain was solubilized with acetic acid and quantified by measuring the absorbance at 550 nm. In the XTT assay, after biofilm formation and washing, the XTT reagent (a tetrazolium salt) was added; metabolically active cells reduced XTT to a colored formazan product, which was then quantified spectrophotometrically at 490 nm. While CV reflects the total biofilm biomass, XTT indicates the viability of the biofilm cells.

### 4.4. CLSM Analysis of Extracellular Matrix

An analysis was performed using *Griffonia simplicifolia* Lectin II (GS-II) conjugated with Alexa fluor 488 dye and a 1 h application to the formed biofilms on the glass slides with 0.5% Triton-X 100 (Sigma-Aldrich^®^, Germany) and 4% formaldehyde. The stained samples were examined using a Leica TCS SPE confocal fluorescence microscope (Leica Geosystems AG, Heerbrugg, Switzerland). The biofilm depth was measured at 4 µm intervals from the bottom of the biofilm along 80 µm with a 10× objective. Finally, the images were processed using FIJI (Image J2, National Institutes of Health, Bethesda, MD, USA). The extracellular matrix density was estimated at the maximum projections of z-stacks. Three fields per sample were obtained, and the extracellular matrix density was calculated as a percentage of the occupied area.

### 4.5. Statistical Analysis

Quantitative variables are expressed as the median (IQR). Continuous variables were compared using a *t*-test in the case of a normal distribution and the Mann–Whitney test in the case of a non-normal distribution. All the statistical tests were 2-tailed. Statistical significance was set at *p* < 0.05 for all the tests. The statistical analysis was performed using IBM SPSS Statistics for Windows, version 21.0 (IBM Corp, Armonk, New York, NY, USA).

## 5. Conclusions

We observed that the FDC activity varies depending on the strain when assessing both the MIC and the MBIC. The best percentage reduction rates of all the biofilm-defining variables were observed for *P. aeruginosa*, whereas the worst results were obtained for *A. baumannii*. Future studies are needed to validate our results in a large sample.

## Figures and Tables

**Table 1:** Characteristics of the strains studied and susceptibility profile of cefiderocol.

Species	Code	Resistance Profile	Origin	MIC (mg/L)	MBIC (mg/L)
***Pseudomonas aeruginosa***	PA1	MDR	Blood	2	2000
	PA2	MDR	Blood	4	2000
	PA3	MDR	Abscess	0.25	128
	PA4	VIM	Blood	>4	8000
	PA5	MDR	Abscess	1	1000
***Acinetobacter baumannii***	AB1	MDR	Abscess	0.5	64
	AB2	MDR	BAL	1	8
	AB3	MDR	PE	2	64
	AB4	MDR	RE	0.5	32
	AB5	MDR	BA	2	16
	AB6	MDR	BA	0.5	16
	AB7	MDR	RE	0.5	64
	AB8	MDR	RE	0.5	64
	AB9	MDR	RE	>4	2000
	AB10	MDR	BA	1	128
	AB11	MDR	Blood	4	8000
***Klebsiella pneumoniae***	KP1	VIM	Blood	1	128
	KP2	KPC	Blood	2	16
	KP3	KPC	Blood	1	64
	KP4	KPC	Blood	0.5	64
	KP5	KPC	Blood	0.25	128
	KP6	KPC	Blood	0.5	8
	KP7	KPC	Blood	0.5	8
	KP8	KPC	Blood	0.5	8
	KP9	VIM	Blood	0.25	8
	KP10	KPC	Blood	0.25	4
	KP11	KPC	Blood	0.5	1000
	KP12	KPC	Blood	0.5	1000

**MDR**, multidrug resistance; **KPC**, *Klebsiella pneumoniae* carbapenemase; **VIM**, Verona integrin-encoded metallo-beta-lactamase; **MIC**, minimum inhibitory concentration; **MBIC**, minimum biofilm inhibitory concentration; **BAL**, bronchoalveolar lavage; **PE**, pharyngeal exudate, **RE**, rectal exudate; **BA**, bronchial aspirate.

**Table 2:** Median (IQR) percentage reduction in log CFU counts, cell viability, biomass, metabolic activity, and extracellular matrix after 48 h treatment of 24 h biofilms with 8 mg/mL of cefiderocol.

Strains	Median (IQR) Percentage Reduction				
	Log CFU/mL	Cell Viability	Biomass	Metabolic Activity	Extracellular Matrix
Overall	67.8 (47.7–80.2)	73.1 (12.4–86.5)	0.0 (0.0–28.9)	59.3 (28.1–97.4)	79.5 (37.3–95.5)
*Pseudomonas aeruginosa*	86.7 (73.8–96.7)	93.2 (86.5–98.0)	47.0 (0.0–82.4)	61.8 (45.6–95.3)	94.7 (85.3–95.9)
*Acinetobacter baumannii*	43.0 (20.7–53.2)	6.1 (0.8–5.0)	0.0 (0.0–11.9)	48.1 (29.8–98.6)	81.5 (55.0–98.0)
*Klebsiella pneumoniae*	75.6 (63.1–80.2)	60.3 (14.3–81.5)	0.0 (0.0–39.4)	56.5 (0.0–95.3)	44.8 (0.2–91.2)

**IQR**, interquartile range; **CFU**, colony-forming units.

**Table 3:** Comparison of the percentage reduction rates for the biofilm-defining variables tested between the 3 species.

Groups	*p*-Value *				
	Log CFU/mL	Cell Viability	Biomass	Metabolic Activity	Extracellular Matrix
PA vs. AB	**0.009**	**0.029**	0.079	0.593	0.282
PA vs. KP	0.082	**0.008**	0.190	0.426	0.05
AB vs. KP	**0.002**	0.054	0.500	0.412	0.148

**PA**, *Pseudomonas aeruginosa*; **AB**, *Acinetobacter baumannii*; **KP**, *Klebsiella pneumoniae*; **CFU**, colony-forming units. * Values in bold represent statistical significance (*p* < 0.05).

## Data Availability

The original contributions presented in this study are included in the article. Further inquiries can be directed to the corresponding author.
